# A Western World Perspective of Survival Benefit of Living Donor Liver Transplantation: A Commentary to the Article by Jackson et al. Published in JAMA Surgery

**DOI:** 10.3389/ti.2022.10931

**Published:** 2022-10-25

**Authors:** Quirino Lai, Jan Lerut

**Affiliations:** ^1^ General Surgery and Organ Transplantation Unit, Department of General and Specialty Surgery, AOU Policlinico Umberto I, Sapienza University of Rome, Rome, Italy; ^2^ Institut de Recherche Clinique et Expérimentale (IREC), Université Catholique de Louvain (UCL), Brussels, Belgium

**Keywords:** intention-to-treat, transplant oncology, survival benefit, hepatocellular cancer, colorectal metastases

Liver transplantation is the best treatment for several liver diseases causing acute or chronic hepatic failure, primary and secondary hepatobiliary tumors, and liver-based inborn metabolic errors (1). Unfortunately, many patients die on the list or are too sick and drop out, thus losing the opportunity to be transplanted (2). Consequently, every effort needs to be made to overcome the allograft shortage.

Recently, the deceased-donor pool has been substantially extended using technical variants like split and domino transplants (3–5), more aged or cardiac death donors, and machine perfusion technology (6, 7). However, all these measures remain insufficient to cover the actual needs.

Living donor liver transplantation (LDLT) represents the best, although ethically more complex, way to overcome allograft shortage. Recently, a study from the US by Jackson et al. published in JAMA Surgery has added relevance to the role of LDLT also in a Western setting (8).

LDLT has many significant advantages. First, LDLT allows transplanting a given patient without harming the patients inscribed on the waiting list (9). Secondly, LDLT consents to offer an “ideal” graft with minimal ischemia time (10). Thirdly, this procedure allows for an electively and timely transplant of a given recipient, therefore offering the best economic solution to cure given liver disease. All these advantages must be counterbalanced with the ethical justification of the procedure and the potential donor risk for morbidity and mortality (11, 12).

Live donation has flourished in Asian centers, mainly due to the historical shortage of deceased donor liver transplantation (DDLT) cases (13). In sharp contrast to the Eastern world, LDLT still represents a (too) limited activity in the Western world based on the challenging balance between the weight of the risks linked to the donor hepatectomy and the benefits to the recipient (14, 15).

This Western hesitation related to LDLT has been “fed” by teams embarked on such programs without having gathered enough experience in transplantation and advanced liver surgery. The too high morbidity rates and some donor mortalities hampered the evolution of LDLT in the Western world, leading in turn to the absence of adequately numbered studies allowing to identify the patient survival benefits. Even worse, the too rapid publication of studies, some of them also presenting methodological flaws, resulted in a negative attitude of the transplant community towards LDLT (16, 17). Thus, the initial enthusiasm was turned into a negative perception.

During the last decade, the safety of LDLT for both donor and recipient has been significantly improved by the Asian transplant centers, focusing on the importance of technical details and liver regenerative physiology (18). The donor risk has been markedly reduced by introducing the concept of technical versatility leading to the most appropriate use of left or right donor graft (19).

The recent US study by Jackson et al. in JAMA Surgery based on the data from the US Scientific Registry of Transplant Recipients (SRTR) refocused the view on the relevance of LDLT (8). Between January 2012 and September 2021, 119,275 liver transplant candidates were analyzed, and only 2,820 (2.4%) received a LDLT. The LDLT group had a significant survival benefit compared to patients remaining on the list. LDLT patients having a Model for End-stage Liver Disease-sodium (MELD-Na) ≥11 had an adjusted hazard ratio for the risk of 1-year mortality of 0.64 (95% CI = 0.47–0.88; *p* = 0.006). LDLT consented to gain 13–17 additional life years according to their different MELD-Na categories. The 13-year survival gain observed in low MELD-Na scores (values 6–10) was particularly appealing.

These results are not in line with previous experiences. A study from the US based on DDLT showed a survival benefit only when the MELD-Na was ≥15 (20). A study about 868 LDLT performed during the period 2002–2009 showed no benefit in patients with hepatocellular cancer (HCC) having a lab-MELD <15 (15).

The Jackson et al. study is the first Western world study confirming that LDLT has the most significant life-saving value with respect to any other curative procedure and that this beneficial effect is faithful also in patients with low MELD-Na, which are more often the patients harboring an HCC.

The field of transplant oncology, a term introduced in literature by our team in 2015, is the most promising field of LDLT (21).

Despite attributing bonus points to HCC patients, many cancer patients still do not get access to a potentially curative treatment in the Western world. Moreover, cholangiocellular cancer and secondary colorectal and neuro-endocrine tumors are not yet fully validated indications for LT (22–25).

This aspect is essential, as primary hepatobiliary cancers are becoming the main indications for LT in many countries. Two recent studies highlighted the importance of LDLT in treating HCC patients.

The monocentric Toronto study (N = 851, LDLT = 25.7%) showed that the 5-year intention-to-treat survival rates were 68% in LDLT vs. 57% in DDLT (*p* = 0.02), and that a potential live donation was a protective factor for death (hazard ratio = 0.67; 95% CI = 0.53–0.86) (26).

The Eastern-Western collaborative HCC-LT effort confirmed this evidence based on the analysis of 13 collaborative centers in Europe, Asia, and North America (N = 3958; LDLT = 31.7%) (27). After balancing the results with a propensity score, LDLT was an independent protective factor that reduced the risk of overall death by 33%–48% in both the international and external validation cohorts. These data indicate that LDLT minimizes the risk of death in HCC patients, mainly by reducing or completely zeroing the risk of drop-out on the waiting list. This effect is even more pronounced if more advanced tumors (i.e., Milan-Out criteria) receive a LDLT. A sub-analysis of this cohort showed that 5-year HCC-related deaths were similar after LDLT and DDLT (12% vs. 12%; *p* = 0.49). Conversely, 5-year HCC-unrelated death rates were markedly superior in the DDLT group (21 vs. 11%; *p* < 0.001), confirming the overall positive effect of LDLT performed in expert centers (28).

The role of LDLT is expected to be also relevant in terms of intention-to-treat survival benefit in the setting of well-selected secondary, colorectal, and neuroendocrine tumors (14, 24–29).

LDLT will allow for a modern oncologic approach in these well selected patients by electively being placed between neo-adjuvant and adjuvant chemotherapies. The Oslo experience with colorectal metastases showed that this approach is feasible and rewarding. The cross-fertilization between LDLT and advanced liver resection technologies has led to the development of the Resection And Partial liver segment 2-3 transplantation with Delayed total hepatectomy (RAPID) procedure, in which a left lobe from a live donor is used (30, 31).

This method may represent a way to substantially extend the number of transplantations for secondary liver tumors without interfering with the waiting list and using a safer approach for the donor. Recently, this technical variant has been successfully applied also in cirrhotic patients (32). The door to a significant extension of LDLT has been opened.

In conclusion, patients receiving a live donation have better survival rates when compared with patients remaining on the waiting list. Additional life-years have been obtained after LDLT in all the classes of MELD-Na severity and the lowest category (MELD-Na 6-10).

LDLT is a very efficacious therapy, especially for well-selected patients with primary and secondary hepatobiliary tumors. The superior intent-to-treat results are mainly due to the planning of elective surgery, thereby eliminating the risk of drop-out on the waiting list. Several technical innovations have been introduced to make live donation safe, and it is expected that this increased safety could lead to a significant role of LDLT in Europe and North America. The Western world should follow the path paved by Asian colleagues for almost four decades. The time has come that US and European centers should embrace LDLT as an option to adopt for curing liver diseases and hepatobiliary cancer patients.

## Data Availability

The original contributions presented in the study are included in the article/Supplementary Material, further inquiries can be directed to the corresponding author.

## Abbreviations

CRLM, colorectal liver metastases; DDLT, deceased donor liver transplantation; HCC, hepatocellular cancer; LDLT, living donor liver transplantation; MELD-Na, model for end-stage liver disease-sodium; RAPID, resection and partial liver segment 2-3 transplantation with delayed total hepatectomy.
